# Emerging Nanomedicine Therapies to Counter the Rise of Methicillin-Resistant *Staphylococcus aureus*

**DOI:** 10.3390/ma11020321

**Published:** 2018-02-23

**Authors:** Alan Hibbitts, Cian O’Leary

**Affiliations:** 1 Tissue Engineering Research Group, Department of Anatomy, Royal College of Surgeons in Ireland, 123 St. Stephen’s Green, Dublin 2, Ireland; cianoleary@rcsi.ie; 2 Trinity Centre of Bioengineering, Trinity College Dublin, 152-160 Pearse Street, Dublin 2, Ireland; 3 Advanced Materials and Bioengineering Research (AMBER) Centre, Royal College of Surgeons in Ireland and Trinity College Dublin, Dublin 2, Ireland; 4 School of Pharmacy, Royal College of Surgeons in Ireland, 123 St. Stephen’s Green, Dublin 2, Ireland

**Keywords:** MRSA, biofilms, nanomedicine, anti-microbial resistance

## Abstract

In a recent report, the World Health Organisation (WHO) classified antibiotic resistance as one of the greatest threats to global health, food security, and development. Methicillin-resistant *Staphylococcus aureus* (MRSA) remains at the core of this threat, with persistent and resilient strains detectable in up to 90% of *S. aureus* infections. Unfortunately, there is a lack of novel antibiotics reaching the clinic to address the significant morbidity and mortality that MRSA is responsible for. Recently, nanomedicine strategies have emerged as a promising therapy to combat the rise of MRSA. However, these approaches have been wide-ranging in design, with few attempts to compare studies across scientific and clinical disciplines. This review seeks to reconcile this discrepancy in the literature, with specific focus on the mechanisms of MRSA infection and how they can be exploited by bioactive molecules that are delivered by nanomedicines, in addition to utilisation of the nanomaterials themselves as antibacterial agents. Finally, we discuss targeting MRSA biofilms using nano-patterning technologies and comment on future opportunities and challenges for MRSA treatment using nanomedicine.

## 1. Introduction

In a recent report, the World Health Organisation (WHO) classified antibiotic resistance as one of the greatest threats to global health, food security, and development [1]. Antibacterial resistance, defined as the reduction or the loss in bacteriostatic or bactericidal efficacy of an antimicrobial agent at doses that would normally exert its therapeutic effect, renders currently-available medications unable to successfully eradicate infection from a patient or animal. As a result, bacterial infections that would conventionally be classified as low-risk or easily-treatable become associated with severe morbidity and mortality. For vulnerable patient populations in the hospital setting, nosocomial infections that exhibit antibiotic resistance can severely complicate management of organ failure [2,3,4], HIV [5], soft tissue infections [6], or intensive care unit (ICU) inpatients [7,8]. Overall, antibiotic resistance doubles the rate of adverse events relative to antibiotic-susceptible infections [9], and ultimately contributes to millions of Euro of hospital expenditure, hundreds of thousands of additional bed days for patients, and thousands of extra deaths [10].

Methicillin-resistant *Staphylococcus aureus* (MRSA) is a micro-organism that is synonymous with antibiotic resistance in the hospital and community setting. Since its first clinical isolation in 1961 [11], MRSA has persisted in hospitals and ICUs, presenting in approximately 40–60% of bacterial isolates, with frequent multidrug resistance [12,13]. Of course, MRSA also presides in the community and animals as a common cause of soft tissue infections and these strains can be diverse in terms of their phenotype, drug resistance patterns, and clinical outcomes [14,15,16,17,18]. MRSA has developed resistance to penicillin- and cephalosporin-based antimicrobials by the acquisition of the *mecA* gene [19]. This results in the expression of an altered penicillin-binding protein subtype PBP2a, for which these agents have reduced binding affinity and a drop in pharmacological action [20]. Additionally, MRSA infections can inhibit the efficacy of antibiotics through horizontal resistance gene transfer from other micro-organisms [21], antibiotic-removing drug efflux pumps [22], and notably for indwelling catheters and other colonized implants, adherent biofilm formation [23,24]. Thus, although a decrease in rates of nosocomial MRSA infections has been observed [25], its widespread presence elsewhere in our environment, numerous bacterial subtypes, and abundance of drug resistance mechanisms remain a significant cause for concern. This is further compounded by the fact that MRSA infection has few clinically approved antibacterial treatments beyond vancomycin and daptomycin, which are two drugs that have their own emerging susceptibility concerns [21,26,27]. Therefore, it is imperative that a new paradigm of antimicrobial therapy is added to our current dwindling arsenal of antibacterial agents.

**Figure 1:**
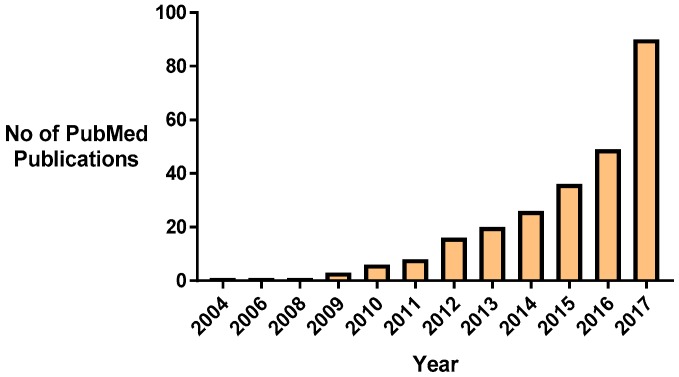
The increasing trend of research into nanomedicines and antibacterial resistance, as reflected by increasing publications in PubMed.

Nanomaterial approaches to combat MRSA antibiotic resistance are one emerging paradigm that can address these challenges [28]. It is particularly promising since the turn of the last decade, research into this area has increased steadily (Figure 1). Nanomedicines, in particular nanoparticles, have the potential to combat antimicrobial resistance by several mechanisms. Nanoparticles themselves can be cytotoxic for bacteria, can enhance the efficacy of current antibiotics by protecting them from detection and degradation, and provide a means of targeted delivery to the microorganisms to maximize the local concentration of agent and bactericidal effect [29,30]. Additionally, the nanopatterning and modification of surfaces and implants at the nano-scale can interfere with bacterial adherence, colonization, and biofilm formation (Section 4). However, despite the wealth of research studies available, few of the promising preclinical studies have translated into clinical trials; this warrants an evaluation of the current state-of-the-art in order to discern the most promising nanomedicines to bring forward to the clinic.

Accordingly, the objective of this review is to summarize the field of nanomedicine therapies for antibacterial resistance to date, with a specific focus on MRSA infection. Firstly, an outline of the some of the most promising therapeutic cargoes of nanoparticles is provided with a description of their various mechanisms of antimicrobial activity. Following this, we discuss the recent developments in utilising nanoparticles as a means of reinvigorating and repurposing previously approved drugs to treat MRSA. For the sake of clarity, we have restricted our review to nanomedicines devoted to eradicating MRSA. While the use of nanoparticles to develop vaccinations against MRSA is a growing field, it has been recently reviewed and well covered by Giersing et al. [31]. Finally, we discuss the use of nano-patterning technologies and surface modification to prevent MRSA biofilm formation and comment on future opportunities and challenges for MRSA treatment using nanomedicine.

## 2. Emerging Therapeutic Agents Used in Nanomedicines to Treat MRSA

### 2.1. Metal Ions (e.g., Silver, Zinc, Gold, etc.)

Metal-containing nanoparticles (NPs) represent a wide field of interest for eradicating or inhibiting the growth of MRSA infections. These NPs can be based on a number of different metal ions with the most common being silver containing NPs (Ag NPs), but also extending to metals, such as gold, magnesium, bismuth, etc. (Table 1). While each metal ion may exhibit specific anti-microbial mechanisms, all share two common anti-microbial effects. Specifically, bacterial cell membrane disruption and reactive oxygen species (ROS) formation. Bacterial membrane disruption occurs when the positively charged ions of the nanoparticle bind to negatively charged parts of the bacterial membrane. This creates pores in the membrane in which cytoplasmic contents flow out of the cell, dissipating the H+ gradient across the membrane which may result in cell death [32,33]. ROS formation then occurs following nanoparticle internalization into the bacterial cytosol which can results in DNA damage and cell death [32,33,34]. It is worth noting that these two anti-microbial mechanisms are equally damaging to eukaryotic membranes and cells, and, therefore, great care must be taken in directing these NPs to their site of action [35].

When applied to MRSA cultures, Ag NPs have been found to be highly efficient in eliminating MRSA colonies in in vitro settings. Specifically, MIC values of between 0.25 and 64.5 μg/mL have been recorded for Ag NPs depending on the NP synthesis conditions and MRSA strains used [36,37,38]. Ag NP performance can also be further enhanced through techniques such as blue light excitation [39]. Ag NPs have also advanced to in vivo testing, mostly as a surface coating on medical implants. In a recent study by Cheng et al., it was found that using Ag NPs to coat titanium implants demonstrated no evidence of live bacteria up to four weeks post-implantation in previously infected animals [40]. Other groups have looked at the in vivo topical administration of Ag NP imbued hydrogels for wound healing. At 15 days post-implantation, it was found that in Ag NPs hydrogel-treated samples, skin exhibited 2.7% of the bacterial count in the control infected but untreated skin. In comparison, silver sulfadiazine cream- and the blank hydrogel-treated groups exhibited 30% and 100% of the bacterial count respectively [41].

Similarly, NPs derived from other metallic sources have also demonstrated potential against MRSA when tested. The majority of metallic ion-based NPs demonstrated strong antimicrobial properties with some studies progressing as far as in vivo trials, and, in many cases, even demonstrating an ability to eliminate MRSA biofilms (Table 1). The efficacy of these NPs has also been reported in numerous studies to be enhanced by controlling the size of NPs [42,43,44], altering pH activity [45], stimulation via ultrasound [43], near-infrared (NIR) stimulation [46], UV stimulation [47], addition of potassium iodide or sodium bromide [47,48], and peptide or antibody conjugation [46,49]. However, not all metal NPs are suitable candidates for treating MRSA, especially so aluminum-containing NPs. Previous studies have found that these NPs may actually increase the potential for drug resistance [50].

### 2.2. Anti-Microbial Peptides and Peptidomimetics

**Figure 2:**
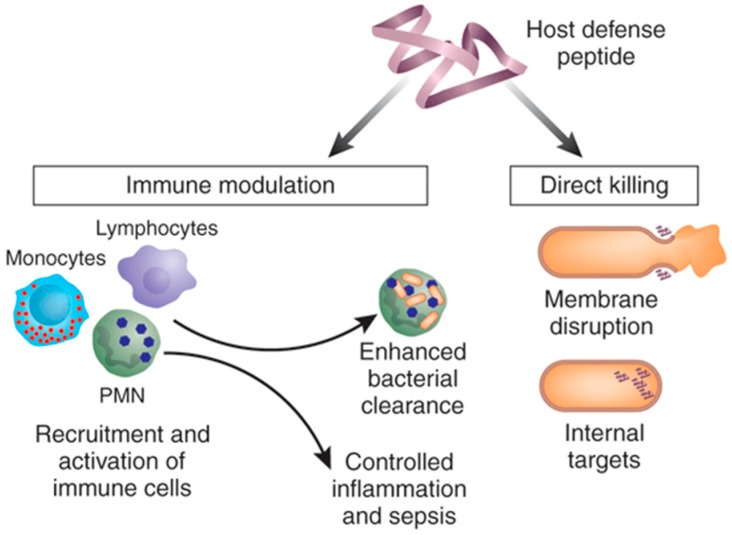
Mechanism of action for cationic anti-microbial peptides; anti-microbial peptides (AMPs) are capable of direct anti-microbial effects and an immune-modulatory effect on the innate immune system, although not all AMPs have both abilities. “Reproduced with permission from [81] published by © Nature Publishing Group.” (2006).

Cationic anti-microbial peptides (AMPs), also known as host defense peptides, represent a diverse field that focus on the use of small peptide fragments to destroy or otherwise disrupt proliferation of pathogenic bacteria. These can broadly be described as being gene encoded, short (10–50 amino acids), with an overall positive charge (generally +2 to +9) and a substantial proportion (≥30%) of hydrophobic residues [81,82]. These characteristics allow for the AMPs to form α-helical, β-sheet, and random coil conformations, and, in conjunction with their positive charge, form the basis for their anti-microbial effect by creating pores in the bacterial membrane [83]. Once AMPs traverse the bacterial membrane through these pores, they are also able to inhibit protein and cell wall synthesis, thereby inhibiting microorganism growth [84]. In addition to their ability to directly kill invading pathogens, AMPs can also function in an immunomodulatory fashion. AMPs are known to mediate the recruitment of immature dendritic cells, by direct chemotactic activity or by upregulation of chemokine production in macrophages, and promote maturation of these dendritic cells directly or indirectly by inducing production of inflammatory cytokines (IL-1b, TNFa) (Figure 2) [85]. In addition, AMPs have also been found to reduce the systemic production of TNFa, IL-1b, and IL-6 [86]. This could allow for a more measured ability to treat MRSA infections locally without the serious concerns of a systemic response or a potentially fatal cytokine cascade in patients.

When AMPs have been applied in attempt to combat MRSA there have been numerous promising findings reported (Table 2, with in-depth AMP reviews available by [85,87,88]). However, in their native state, AMPs have been limited in their development due to their poor stability and activity at physiological conditions coupled with their vulnerability to protease degradation, potential immunogenicity, and cytotoxicity to red blood cells [89,90]. To address this, AMPs are now being developed with modified peptide sequences to enhance activity [84,91,92] or are being synthesized as pro-drugs or “peptidomimetics” to avoid toxicity issues, enhance retention, and allow for improved efficacy at the site of action. Several strategies exist to achieve this, including modifying the carbon chain length and functional group [90], PEGylation, net charge reduction [93], nanoparticle or antibody conjugation [94,95], and synergistic delivery with or antibiotics [96].

### 2.3. Oligonucleotides (e.g., RNAi, TFD, CRISPR, Aptamers)

While the research of oligonucleotide delivery and RNA interference (RNAi) in eukaryote cells for therapeutic applications is a well-established field, it is now also being considered for improving outcomes in bacterial infections, such as MRSA. While less widely reported, this strategy has in fact demonstrated potential to reduce bacterial growth from as early as 2003 [103] in *Escherichia coli* (*E. coli*) and 2006 for MRSA [104]. This is in spite of the obvious challenges that are posed by the need to deliver large negatively charged nucleic acids across both the cell wall and membrane of gram-positive bacteria, such as MRSA.

Initial testing against MRSA has focused on the more established RNAi systems of siRNA and miRNA in attempts to down-regulate expression of proteins critical to bacterial proliferation or virulence. In their 2006 study, Yanagihara et al. found that MRSA would internalize siRNA without nanoparticle assistance and was capable of reducing expression of the virulence-associated protein staphylocoagulase by up to 40% in vitro. This was also investigated in a murine model of haematogenous pulmonary infection, whereby prior-incubation of the bacteria with anti- staphylocoagulase siRNA significantly reduced growth by 1 log cfu/mL [104].

While this study demonstrated that siRNA could be spontaneously internalized into MRSA, it is unlikely that this reflects a viable method of treatment due to the poor serum stability and rapid clearance in the body of free nucleic acids. Many of the more recent studies have focused on the potential of oligonucleotides and RNAi to re-potentiate current antibiotics by targeting resistance genes. This has been shown to be possible in studies by Meng et al. using lipidic carriers to deliver antisense oligonucleotides targeted to the *mecA* gene. This gene is known to play a role in β-Lactam resistance in MRSA and, by targeting it, it was found that it was possible to restore MRSA susceptibility to oxacillin in vitro and in vivo [105,106,107].

**Figure 3:**
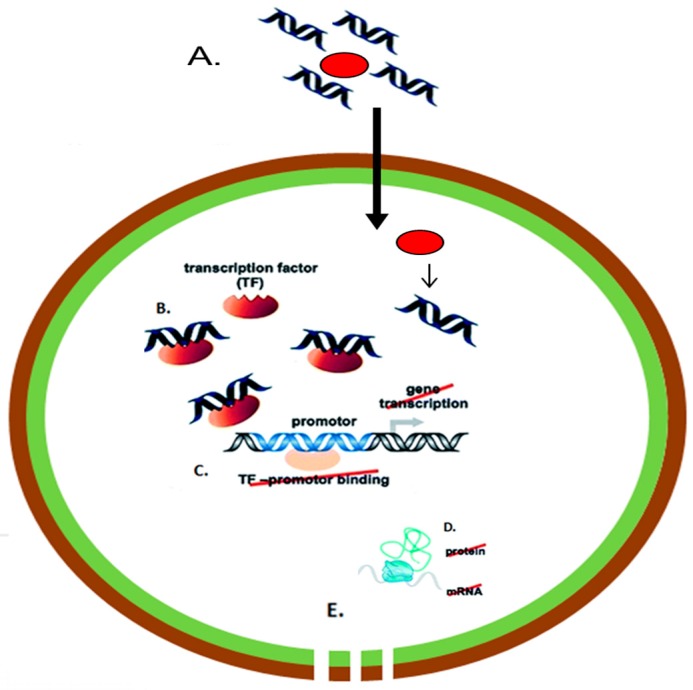
Schematic of nanoparticle-transcription factor decoys (TFD) mediated treatment of MRSA (**A**) administration of nanoparticle-TFD nanocomplexes to bacteria; (**B**) TFD release in cytoplasm and binding of transcription factor; (**C**) inhibition of transcription; (**D**) failure to produce critical proteins; (**E**) bacterial inhibition. “Reproduced with permission from [111] published by © Royal Society of Chemistry.” (2014).

A potentially more robust method of directly inhibiting MRSA growth using nucleic acids is also being investigated using transcription factor decoys (TFDs). TFDs are short double-stranded DNA molecules containing a specific transcription factor binding sequence in the promoter region of the gene of interest (or the sequence can also match the consensus DNA recognition motif of a target transcription factor in the genome) [108]. On delivery through the bacterial cell wall, TFDs competitively inhibit gene expression by sequestering transcription factors, and thus reduce protein expression. This is particularly attractive as TFDs can be targeted towards highly conserved promotor regions controlling processes, such as cell wall metabolism (Figure 3). This has the dual benefits of reducing potential resistant mutations as well as eliminating the potential for off-target effects in humans. TFDs have been successfully delivered in studies using *E. coli* and *Clostridium difficile* (*C. difficile*), but at the time of writing, have yet to be established in MRSA cultures [109,110].

The remarkable gene editing abilities of the CRISPR (clustered, regularly interspaced, short palindromic repeats)/Cas9 (CRISPR-associated protein 9) are now also being applied to overcome the spread of MRSA. As a brief overview, CRISPR-Cas is an endogenous system that is derived to protect bacteria and archaea from foreign genetic elements, such as plasmids or bacteriophages. CRISPR-Cas system consists of two general components: CRISPR RNAs (crRNAs) and Cas proteins. The crRNAs base pair with complementary DNA or RNA sequences associated with an invader, and the Cas proteins clear the recognized genetic material [112]. Subsequently, it has been found that a single protein, Cas9, could be harnessed for site-specific DNA binding and cleavage [113,114].

This extremely accurate method of gene editing has now been applied as an anti-MRSA strategy in a study by Bikard et al. Using a delivery system known as a “phagemid”, whereby the *cas9* gene and its RNA guide/s sequences were incorporated into plasmid and packaged in a bacteriophage capsid, it was possible to deliver the gene editing machinery to the MRSA with a high degree of transfection efficiency. By encoding the *Streptococcus pyogenes* (*S. pyogenes*) *cas9*, tracrRNA and designed CRISPR array it was possible to elicit a 10^4^-fold reduction in the number of viable colonies in vitro. Phagemids were also capable of specifically targeting resistant strains in a mixed population by incorporation of the *aph-3* kanamycin resistance gene. When tested in vivo in a mouse skin colonization model containing a mixed population of kanamycin-resistant and kanamycin sensitive *S. aureus* it was found that the phagemid targeting the *kanR* gene reduced the proportion of kanamycin-resistant *S. aureus* [115].

Finally, one possible strategy to avoid oligonucleotide delivery challenges has been to utilize oligonucleotides as targeting ligands as opposed to direct therapeutics to enhance the selectivity of other anti-microbial nanomedicines. Aptamers are single-stranded nucleic acids (RNA or DNA, 20–100 nucleotides) developed in vitro to perform a specific function, usually specific protein binding. Aptamers are normally generated using the SELEX (Systematic Evolution of Ligands by Exponential Enrichment) method. This involves testing large libraries of oligonucleotides of approximately 10^14^–10^15^ configurations against a target protein. Following this, iterative rounds of selection-amplification cycles are utilized to enrich the populations with high protein binding potential [116]. Aptamers possess high physical and chemical stability, low immunogenicity, and easier to mass produce when compared to traditional antibodies [117].

Aptamer targeting has previously been applied to improve MRSA detection and diagnosis [118,119] and is now being investigated for aiding direct intervention against MRSA infections. In a recent study, Ocsoy et al. [120] demonstrated that by conjugating DNA aptamers specific to gold nanorod particles they were capable of inactivating 95% of MRSA following NIR stimulation. In comparison, there was no discernable reduction in MRSA activity using aptamer-free Au nanorods treated under the same conditions [120]. This treatment strategy was also described using iron magnetic core-gold plasmonic shell nanoparticles attached with an MRSA-specific aptamer with efficient MRSA clearance achieved in infected whole blood samples [121].

## 3. Nanoparticle Delivery of Antibiotics “Old Drugs, New Tricks”

With the timeline and cost of development for novel therapeutics now estimated at up to $2.6 billion over 11 years [122], increasing attention is now being given to the possibility of reinvigorating or repurposing previously approved molecules to treat MRSA. This can take two approaches; the first relies on the screening of libraries of approved drugs (or drugs that made it to clinical trials, but ultimately failed to receive regulatory approval) in order to identify candidates that can be repurposed as treatments for MRSA. The second approach focuses on the potential use of nanoparticles to improve the therapeutic profiles of previously approved antibiotics for MRSA with the primary aim of both being a shorter regulatory approval process and quicker route to market. In keeping with the nanomedicine focus of this review, only enhancing SMDs through nanoparticle encapsulation will be considered here. For a full review regarding the challenges in repurposing SMDs for MRSA treatment, see the recent review by Thangamani et al. [123].

### 3.1. Chitosan

Chitosan is a linear polysaccharide, derived from the deacetylation of naturally occurring chitin (normally obtained from crustaceans or fungi), and consists of d-glucosamine and *N*-acetyl-d-glucosamine units linked by β-1,4-glycosidic linkages [124]. Chitosan is an especially attractive candidate for nano-encapsulation of drugs as it has been found to exhibit its own antimicrobial effects through its positive charge (and accordingly its ability to disrupt the bacterial cell wall) in a wide range of organisms such as algae, bacteria, yeasts, and fungi [125]. Studies have found that even the addition of free chitosan in solution can have a beneficial and synergistic effect against MRSA and biofilms when co-delivered with selected antibiotics (Table 3) [126]. In addition, chitosan is especially attractive when considering recent findings that indicate that MRSA is more susceptible to inhibition than methicillin-sensitive strains [127]. This activity known to be influenced by various factors including pH, microorganism species, presence or absence of metal cations, pKa, Molecular weight (Mw), and degree of deacetylation (DD) of chitosan [125].

When considering the potential for a synergistic effect with antibiotics, it has also been found that forming chitosan into nanoparticles can further increase their efficacy. In an early study by Qi et al. [128], it was found that the MIC of a 220 kDa chitosan solution against *S. aureus* (methicillin susceptible strain ATCC 25923) at pH 5 has been found to be 8 µg/mL. However, when formed into a nanoparticle and no co-administration of antibiotics, the antimicrobial effect was found to decrease to <0.25 µg/mL, owing to the increased surface area and charge density of the nanoparticle structure [128]. (Although, when considering that other publications have reported chitosan MICs in the mg/mL range [126,127,129], it is possible that this reporting of 8 µg/mL and subsequent reductions in MIC may be typographical error). Building on the advantages offered by co-delivery with antibiotics and nano-encapsulation in chitosan it has been found that it was possible to elicit strong anti-microbial effects using previously ineffective antibiotics using this strategy [130,131,132,133]. In other studies, the improvement to the antibiotic’s MIC was less pronounced [134], but advantages were seen when assessed against intracellular bacteria [135]. Furthermore, it was also possible to tailor the release rate of antibiotic using metallic-chitosan nanoparticles; however, this may have an adverse effect on the MIC of the antibiotic [136,137,138].

### 3.2. Liposomes and Solid Lipid Nanoparticles

**Figure 4:**
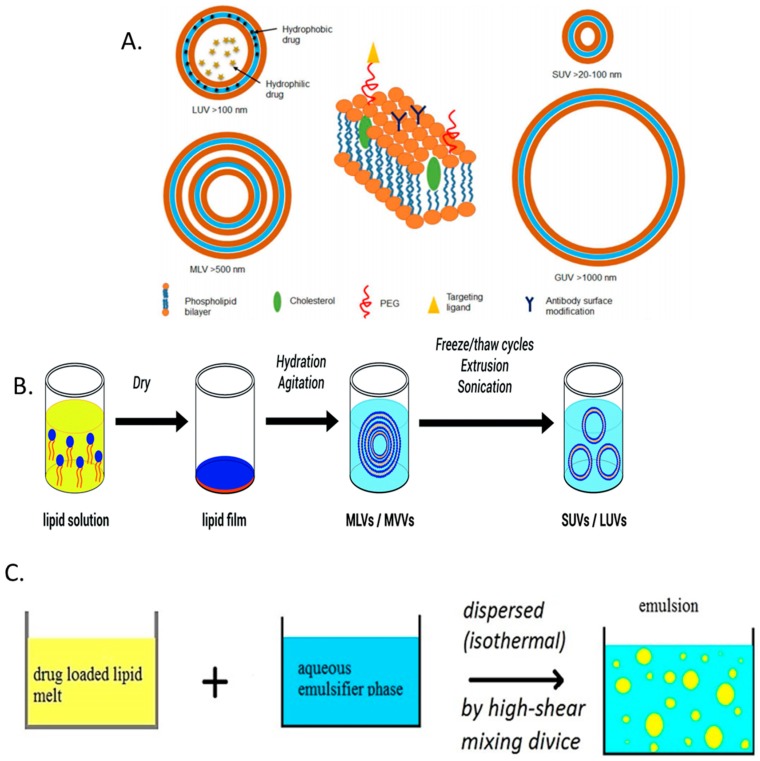
(**A**) Various conformations and constituents of liposomal formulations. Clockwise from left, large unilamellar vesicles (LUV), small unilamellar vesicles (SUV), giant unilamellar vesicles (GUV) and multilamellar vesicles (MLV); (**B**) liposome formulation via the thin-film hydration method; and (**C**) solid lipid nanoparticles (SLNP) synthesis using the hot homogenization technique of organic and aqueous phases. “Reproduced with permission from [139,140,146] published by © American Chemical Society (2015), Royal Society of Chemistry (2016), Pharmaceutical Society of Japan (2015).”

While there are numerous conformations, liposomes can generally be described as spherical vesicles consisting of at least one amphiphilic lipid bilayer with an internal aqueous core. The lipid bilayer can be further augmented with additional components, such as cholesterol or poly ethylene glycol (PEG), in order to improve stability or biological retention [139] (Figure 4A). Liposomes are commonly produced using thin-film hydration, whereby the lipid components are dissolved in an organic solvent (along with any hydrophobic drugs to be delivered). The solvent is then evaporated by rotary evaporation followed by the application of an aqueous solvent to rehydrate the film. Addition of a hydrophilic drug at this point allows for its encapsulation as the lipid film is being rehydrated (Figure 4B). Additional methods for liposome synthesis may also include reverse-phase evaporation, freeze-drying, and ethanol injection [139,140]. Subsequent techniques, such as membrane extrusion, sonication, homogenization, and/or freeze-thawing are then use to control the size and size distribution of the liposomes. Liposomes present an attractive means of drug delivery due to their flexibility in size, composition, charge, lamellarity, and their pre-existing record of clinical approval; however, they are also hindered by a number of weaknesses. These include potential cytotoxic effects, poor stability and unwanted burst drug release, batch to batch reproducibility, and low drug entrapment [139,140].

In an effort to overcome some of the difficulties in developing drug loaded liposomes, researchers are also investigating solid lipid nanoparticles (SLNPs). SLNPs emerged as an evolution on early nano-emulsion approaches, whereby poorly water-soluble lipophilic drugs were incorporated into lipid droplets for drug delivery [141]. For the formation of SLNPs, the oil of the fat emulsion is replaced by a solid lipid or a blend of solid lipids or wax, which therefore makes the lipid core of the SLNP solid at room and body temperature. SLNPs are composed of 0.1–30% weight/weight (*w*/*w*) lipid dispersed in an aqueous solution of 0.5–5% (*w*/*w*) surfactant as stabilizing agent (Figure 4C). The size and physicochemical properties of the SLNPs is readily tunable depending on the lipids and surfactants used [142,143,144]. When compared to liposomes, SLNPs possess high drug stability and prolonged release, and can be formulated using materials that have regulatory approval [143,145]. However, care must be taken to ensure the correct selection of drug to be incorporated. Owing to their lipidic core, drugs with poor miscibility in organic solvents are unlikely to give high encapsulation efficiencies, which can reduce the availability of antibiotics to choose from.

Liposomes and solid lipid nanoparticles carriers represent a highly appealing nanomedicine platform for antibiotics delivery as one of the few nanocarriers that have gained clinical approval for a wide variety of drugs and indications [147]. While the majority of these have been for the delivery of chemotherapeutics, some of the earliest approved drugs were Abelcet^®^ (1995) and Ambisome^®^ (1997), as released by Sigma-Tau Pharmaceuticals and Astellas Pharma, respectively. These are both formulations of the anti-microbial Amphotericin B for the treatment of severe and invasive fungal infections [147]. The development and approval of these nanomedicines is especially promising for the development of MRSA treatments since they demonstrate similar challenges as fungal infections, such as Candidiasis. Specifically, both of the infections pose a challenge due to the presence in each microbe of a gram-positive cell wall, local and systemic infections, and biofilm formation [148,149].

Although no liposomal/SLNP-antibiotic drugs are currently on the market for MRSA treatment, the potential has been investigated as early as 1994. In this early publication, Onyeji et al. found that liposome-encapsulated vancomycin was readily internalised by infected primary human macrophages. Furthermore, intracellular MRSA was significantly (*p* < 0.001) reduced following treatment with the encapsulated vancomycin [150]. In more recent years, other researchers have built on these findings and have been successful in progressing to in vivo models. Using the thin-film hydration method, Sande et al. encapsulated vancomycin and reported a 2–4× improvement in MIC values depending on the liposome formulation used and MRSA strain tested. On in vivo testing in a mouse model of systemic MRSA infection, liposomal vancomycin demonstrated significantly enhanced MRSA clearance versus PBS controls (*p* < 0.001) in the kidneys and spleen, and was significantly improved versus free vancomycin controls in the kidney (1 log, *p* < 0.015) [151]. It should also be noted that as a preliminary study there were no pharmacokinetics or histology reported, and, as such, any potential benefits to pharmacokinetics or organ toxicity (specifically vancomycin-associated nephrotoxicity) may have been overlooked. Subsequently, studies have investigated the possibility of enhancing the effect and residency time of vancomycin liposomes using PEGylation. In studies examining intracellular MRSA in alveolar macrophages, it was found that while non-PEGylated liposomes loaded with vancomycin were capable of significant improvements in MRSA clearance, PEGylated liposomes had no significant impact. However, when tested in healthy mice, it was found that both PEGylated and non-PEGylated liposomes significantly increased residency times as compared to free vancomycin. Significantly higher levels of PEGylated liposomes were also found 24 h post-administration in the lung compared to non-PEGylated [152,153]. Additional in vivo studies have further demonstrated that liposome encapsulation of vancomycin reduces accumulation in the kidneys, which has direct implications in avoiding vancomycin-associated nephrotoxicity [153,154].

Aside from the encapsulation of vancomycin, several other strategies involving liposomes have been investigated. Various other, less commonplace, anti-microbial agents for encapsulation have also been examined including chloramphenicol [155], azithromycin [156], oleic acid [157], and cinnamon oil [158]. These have been found to perform well up to studies involving biofilms and in vivo efficacy [157,158]. Liposome-antibiotic delivery was further enhanced in other cases through the addition of other molecules previously described in this review, such as chitosan [159] or anti-microbial peptides [156].

Similarly, SLNPs have also demonstrated significant promise when used to deliver anti-MRSA antibiotics. While it might be assumed that, due to vancomycin’s extremely hydrophilic nature, it is not a ready candidate for encapsulation into the lipid core of a SLNP; strategies have been developed to achieve this. SLNP encapsulation of vancomycin was obtained by the ion pairing of triethylamine neutralized vancomycin with a lipophilic contra-ion (linoleic acid). Using this technique, it was possible to formulate highly stable SLNPs capable of exerting an antimicrobial effect for up to 54 h (vs. 18 h for free drug) [160]. This technique is also of additional interest as it also utilizes the enhanced microbial effect of linoleic acid nano-emulsions that have been reported in other studies [161]. Vancomycin was also encapsulated in SLNPs by the same group using an acid cleavable lipid that allowed the creation of pH-responsive SLNPs. These particles gave a 22-fold improvement in MRSA clearance in a mouse skin infection model when compared to drug only controls and allowed for site-specific targeting [162]. As with liposomes, there are also numerous studies available that now detail a combined approach to delivering anti-MRSA antibiotics. Some of which include the incorporation of dendrimers [163], naturally occurring polymers [164] and antimicrobial metallic ions [165]. Most significantly, using a nano-emulsion of vegetable oil and water with surfactants and alcohol identified as NB-201, Cao et al. were able to demonstrate anti-MRSA efficacy (as well as a reduction in pro-inflammatory cytokines) in mouse skin abrasion models and progressed as far as a porcine model of infected wounds [166].

### 3.3. Synthetic Polymer Nano-Carriers

In addition to the naturally derived polymers of chitosan and lipidic nanoparticles, there are a wide variety of synthetic nanoparticles that are also currently being investigated for their ability to deliver antibiotics against MRSA. One of the most commonly researched are nanoparticles based on poly(lactic-*co*-glycolic acid) (PLGA) due to its well established character and success in achieving FDA approval [167]. Frequently, drug encapsulation in PLGA nanoparticles utilizes a single- or double-emulsion protocol whereby the drug is first dispersed in the aqueous phase containing hydrophilic surfactants. Following this, it is then emulsified in polymeric solution dissolved in an organic solvent in the presence of lipophilic surfactant, which forms the single water-in-oil (W1/O) emulsion. When forming the double emulsion the primary emulsion is emulsified in the second aqueous phase containing stabilizers, with polyvinyl alcohol commonly used. The mixture is then homogenized by high shear homogenizer or sonication. This process leads to the formation of double or water in oil in water (W1/O/W2) emulsion. In the last stages of formulation, the organic solvent is evaporated to precipitate the nanoparticles, which are then dried and stored [167]. This represents a well-established formulation strategy but one of the drawbacks to PLGA nanoparticles is that they are highly sensitive to any changes in their formulation conditions e.g., surfactants/solvents used, homogenization method, molecular weight of starting material, etc. [167,168].

Drug selection is one of the most influential of these variables and for their use in the treatment of MRSA can be the cause of poor test outcomes. Specifically, in the case of vancomycin and similar drugs, their highly hydrophilic nature can present difficulties in achieving a workable encapsulation efficiency for PLGA nanoparticles. This was recently highlighted by Ritsema et al. using a variety of different glycolic acid-based block co-polymers and either vancomycin (freely soluble in water, 225 g/L) or bedaquiline (close to insoluble, 192 μg/L). Clear differences were observed where % (*w*/*w*) drug loading of 8–11% was possible for either drug, encapsulation efficiencies for the highly hydrophobic bedaquiline was 95–99%, whereas vancomycin was only capable of a encapsulation efficiency of 26–32% [169]. While this presents a challenge in developing an effective therapy, several groups are now reporting innovative methods of enhancing the outcomes of PLGA-antibiotic nanoparticle treatment.

**Figure 5:**
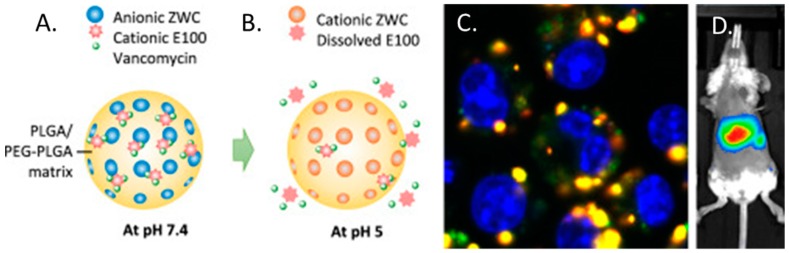
(**A**) PpZEV composition and behavior at pH 7.4 and (**B**) degradation and release at pH 5; (**C**) Uptake of fluorescently labelled vancomycin and PpZEV by MRSA-infected macrophages with vancomycin stained green; PLGA stained red and cell nuclei stained blue and (**D**) whole body imaging of BALB/c mouse that received DiR-loaded PpZEV nanoparticles by intravenous injection. (Redrawn with permission from [171].)

In one such study, Che-Ming et al. reported a 2–4% (*w*/*w*) vancomycin loading efficiency; however in creating platelet membrane-cloaked PLGA nanoparticles, they reported significantly higher levels of MRSA clearance in vitro compared to free drug. Furthermore, in a mouse model of systemic MRSA252 infection, cloaked nanoparticles demonstrated significantly better antimicrobial efficacy in the liver and spleen and was at least as effective in the blood, heart, lung, and kidney compared against free vancomycin at six-fold the dosage [170]. Research has also been undertaken to develop PLGA particles that have demonstrated a pH responsive release profile and enhanced intracellular targeting. This formulation was named “PpZEV” and consisted of several distinct components, each serving a particular purpose. (i) PLGA (P), constituted the overall delivery vehicle; (ii) PEGylated PLGA (p) was included to improve solubility and vancomycin release; (iii) Eudragit E100 (E) (a copolymer of dimethylaminoethyl methacrylate/butyl methacrylate/methyl methacrylate) to improve vancomycin encapsulation; and (iv) a chitosan derivative called ZWC (Z) to trigger pH-sensitive drug release (Figure 5A,B). When tested, it was found that vancomycin encapsulation rates were improved from 3.8% (*w*/*w*) for unmodified PLGA particles up to 8.3% (*w*/*w*) in the final PpZEV formulation. PpZEV nanoparticles also displayed increased drug release at pH 5 (mimicking intracellular delivery) and significantly higher levels of uptake and MRSA clearance in infected macrophages when compared to free vancomycin and unmodified PLGA-vancomycin particles (Figure 5C). While no efficacy was tested in vivo, biodistrubtion demonstrated an organ specific persistence in the liver and spleen (the organs of interest) of mice for up to 96 h (Figure 5D) [171]. In other studies, proof of concept using PLGA for vancomycin delivery has progressed as far as rabbit models of MRSA-associated infective discitis with local, intra-discal administration of PLGA-vancomycin demonstrating superior bacterial clearance with a lower relative dose of antibiotic [172].

In addition to PLGA based nanocarriers for antibiotic delivery, other synthetic polymers investigated have included poly-ε-caprolactone (PCL), polyacrylate (PA), poly (methacrylic acid) (PMAA) and novel polymers specifically designed for anti-microbial delivery. In the case of PCL polymers, particles have so far been formulated to encapsulate both vancomycin and chloramphenicol with a drug loading of 5% (*w*/*w*) and encapsulation efficiency of 98.3% reported respectively. PCL-antibiotic particles were tested for activity in an in vivo burn-wound mouse model and a rabbit model of osteomyelitis where they were observed to outperform free drug controls in both models [173,174]. Similarly, promising results were also observed using PA or PMAA-based nanoparticles which were then used to anchor antibiotic molecules. This strategy has been investigated as a means of re-potentiating penicillin and in initial studies it was found that it was possible to maintain the potency of penicillin while increasing its resistance to stability toward β-lactamase [175,176]. However, one possible drawback to this method of antibiotic delivery is that, due to the covalent bonds used to immobilize the penicillin to the nanoparticle, the chemical structure of the drug is effectively altered. Thus, it may be viewed as a “new chemical entity” (NCE) or a pro-drug in regulatory terms and may therefore require a full cycle of clinical trials prior to approval and use in the clinic.

Finally, Amato et al. have recently reported a novel approach based on a pro-antimicrobial polymer network comprised of degradable acetals (PANDA). Using this strategy, the antimicrobial agent p-anisaldehyde (pA) was entrapped in a polymeric mesh that was capable of controlled release under acidic conditions. When released it was observed that the antimicrobial activity of the pA was retained and resulted in significantly improved inhibition of MRSA compared to free drug controls with minimal toxicity observed [177]. However, it should be noted again, the potential for additional regulatory hurdles that may be associated with chemically binding the active drug to the delivery vector.

## 4. Nanomedicines and MRSA Biofilms

### 4.1. Overview of Biofilm Formation

Biofilms are a specific mechanism of MRSA persistence and antibacterial resistance for which nanoscale approaches can offer a novel means of microbial eradication. Biofilms are aggregates of microorganisms in which the bacteria are encased in a self–produced protective matrix of extracellular polysaccharide that are highly adherent in nature (reported in [178]). In addition to this matrix acting as a defensive barrier that metabolizes drug molecules or reduces permeation [179], embedded bacteria adopt a different proliferative profile from planktonic bacteria. In this state, bacteria are more quiescent, culminating in a reduction in the activity of most conventional antimicrobial agents that target dividing cells [180]. In this form, MRSA can persist on indwelling devices such as catheters in high-risk patients with resistance to vancomycin [181], or even within cells and tissue [182]. The peri-operative colonization of orthopedic implants is of particular concern within the hospital setting [183,184,185]. Similar to their promise as systemically delivered antibacterials, surface-coating nanoparticles can also be applied as a novel means to treat biofilm-associated infection. Additionally, the application of nanoscale modifications to device surfaces can be implemented to specifically target biofilms. However, in order to identify the processes by which nanomaterial technologies can prevent MRSA biofilm formation or stimulate biofilm destruction, it is important to first have an understanding of the key elements of its generation.

In general, four stages are involved in the life cycle of a biofilm [180,186]: bacterial adherence to a substrate, early micro-colony proliferation and extracellular polysaccharide matrix production, biofilm maturation and bacterial release. Bacterial adhesion can involve reversible and irreversible interactions, primarily mediated by adhesin molecules on the surface of the bacterium [187]. The production of the extracellular shield in MRSA occurs as a result of upregulation of pro-biofilm genes such as *fnb*, *agr*, *sarA*, and *icaADBC* [182,188]. Therefore, nanomaterial approaches that prevent initial adhesion, provide local bactericidal action in high concentrations with possible controlled release of drug, silence biofilm genes, improve drug penetration, or degrade the extracellular barrier can all specifically target these processes. In this section, we will focus on the the nanopatterning of surfaces and the coating of implantable surfaces with nanomedicines as examples of nanomedicine applications. For a more extensive review on disrupting biofilms through cellular pathways or targeting polysaccharide matrix composition, the reader is referred to an excellent review from Koo and colleagues [180].

### 4.2. Nanopatterning & Surface Topography

**Figure 6:**
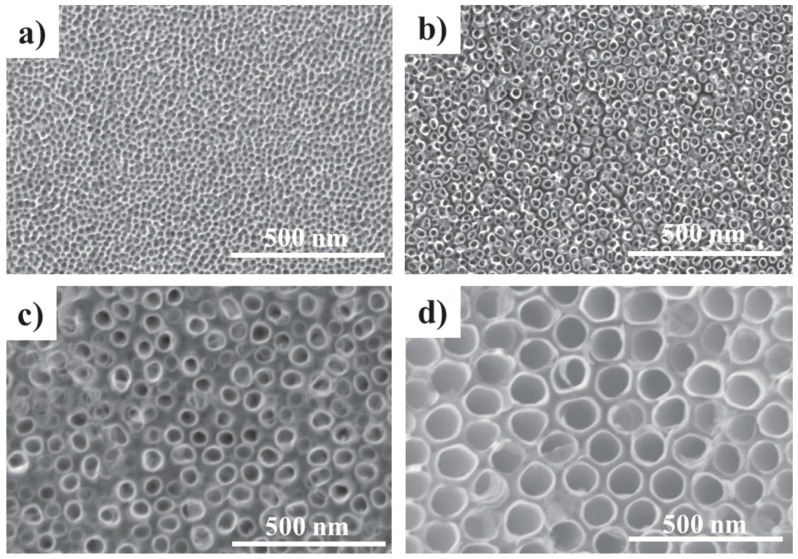
TiO_2_ nanoscale surface modifications. Representative scanning electron micrographs of (**a**) 15 nm nanopores; (**b**) 15 nm nanotubes; (**c**) 50 nm nanotubes; and (**d**) 100 nm nanotubes. “Reproduced with permission from [189] published by © IOP Publishing.” (2015).

In the simplest sense, the most logical solution to prevent biofilm formation on medical device surfaces is to design them so that bacterial adherence is significantly impaired. Many orthopedic implants are titanium-based in nature and nanostructured titanium can increase protein adsorption, host cell attachment, and integration into tissue (Figure 6, [189]). Conversely, nanoscale alterations to surfaces can also significantly influence *S. aureus* attachment and biofilm matrix production [190]. Bacteria such as MRSA respond to surface characteristics such as roughness, hydrophobicity, and surface charge [191]. Indeed, in addition to increasing host cell attachment, commercially-pure titanium and nanotubular titania surfaces have also been found to be adherent for staphylococcal microorganisms, albeit with a higher degree of dead cell attachment when fluorine ions are involved in the fabrication process [192,193]. On the other hand, more irregular nanorough patterns experienced less bacterial attachment, but a higher degree of cell viability on their surface. Moreover, the grade and polishing of the titanium bulk itself can also influence bacterial activity [194]. Surface chemistry also has a role to play in microbiological response. For example, Foka and colleagues demonstrated that the presence of different functional groups, in the presence or absence of dynamic fluid flow analogous to what bacteria experience in vivo, influence extracellular polysaccharide production and *ica* gene expression in *Staphylococcus epidermidis* (*S. epidermidis*) [195]. Taken together, these studies emphasize the importance of appropriate nanoscale design of medical implants and the potential to modulate MRSA attachment and the incidence of biofilm contamination.

There are many technologies and methods available to pattern device surfaces at the nanoscale level that are physical or chemical in mechanism, ranging from photolithography to micro-contact printing (reviewed in [196]). These options provide the opportunity to develop a series of novel bioinspired nanopatterns that can augment the anti-adherent and bactericidal efficacy of device surfaces. Two recent studies by Diu et al. and Bhadra et al. illustrate this approach well, where nanopatterns based on insect wings have been mimicked upon titania surfaces [197,198]. The first study by Diu et al. used the nanopillar topography of cicada wings [199] as a template to fabricate titania nanowire arrays. Interestingly, this study found that topographical design had a selective bactericidal effect for motile bacteria but had no toxic effect on motile bacteria. Similarly, dragonfly-inspired topography was also selectively toxic for motile bacteria, such as *Pseudomonas aeruginosa* [198]. These studies explain such results by postulating that motile bacteria are more readily pierced and damaged by the nanopillar structures as they migrate, and that Gram positive cells might be more resistance to physical penetration on account of their thicker peptidoglycan cell wall. Of course, as a relatively non-motile microorganism, these designs would be ineffective against MRSA. However, it is clear that nanopatterned surfaces can operate as physically bactericidal agents, and although the specific design that is required for MRSA eradication or biofilm is currently unknown, new anti-MRSA nanoscale patterns in the future could provide another means of eliminating the drug-resistant bacteria.

Accordingly, this point highlights the most attractive characteristic of nanopatterning as bactericidal or anti-biofilm agents—the reduced potential for drug resistance. Physical mechanisms of bacterial death and reduced adherence would theoretically avoid current mechanisms of MRSA resistance (Section 1), and in contrast to therapeutics for which resistance can develop, or that have a finite drug quantity incorporated into an implant’s surface, any antibacterial activity would persist until the surface or device is biodegraded. However, as the studies above highlight, our current understanding of the nanoscale modifications that are required to target MRSA are currently unclear, resulting in most of the research focusing on the incorporation of nanomedicines onto device surfaces.

### 4.3. Nanoparticle Surface Treatment

The treatment of implant surfaces with nanoparticles can prevent biofilm formation primarily by facilitating maximum MRSA-therapeutic exposure prior to biofilm formation. As described in previous sections, metallic nanoparticles are bactericidal in their own capacity, while other polymeric nanoparticle formulations generally function as reservoirs for sustained antibiotic release or as vectors to chaperone therapeutics into the cell, such as with nucleic acid molecules or other agents that have intracellular activity. In conjunction with the principal nanoparticles that have been explored in Section 2 and Section 3, most of the studies into surface treatment with nanomedicines have focused on the incorporation of silver nanoparticles (Ag NPs) into orthopaedic medical devices to prevent staphylococcal osteomyelitis (Table 4).

**Figure 7:**
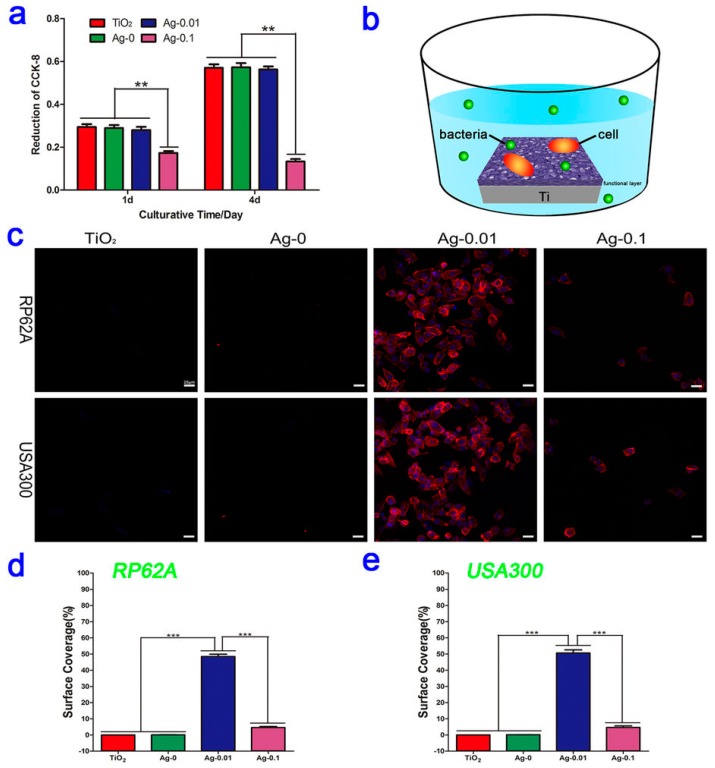
(**a**) HT1080 fibroblast cytocompatibility cultured on micro-arc-oxidized TiO_2_ coatings with and without Ag doping. ** *p* < 0.01 vs. the Ag-0.1 group; (**b**) Schematic illustration of the co-culturing process for the fibroblasts, bacteria and samples; (**c**) Fluorescent images of fibroblast cells on four different specimens contaminated with *Staphylococcus aureus* (PR62A) or *Staphylococcus epidermidis* (USA300) strains after staining with DAPI (blue) and TRITC-phalloidin (red); (**d**,**e**) The corresponding surface coverages of the four samples contaminated with RP62A or USA300. *** *p* < 0.001. “Reproduced with permission from [51] published by © Nature Publishing Group.” (2016).

Ag NPs are without doubt the most widely investigated antimicrobial nanomedicine for the coating of implanted orthopedic devices, either alone or in composite form. Due to the aforementioned toxicity concerns of systemically administered Ag NPs [200], local release of Ag^+^ ions can avoid adverse effects, while retaining therapeutic activity against biofilms. Many orthorpedic implants are titanium-based in nature and nanostructured titanium can increase host cell attachment and integration into tissue [190], prompting the interest for silver NP coating. The coating of titanium materials can be performed using a variety of methods, including silver solution coating and drying with or without ultraviolet light catalysis or oxidation [40,201,202], plasma immersion ion implantation [203], and plasma electrolytic oxidation-fabricated titania coating with subsequent hydrothermal coating of Ag NPs [51]. These methods typically incorporate a reduced form of silver into the material surface that can be oxidised to elute biologically active cations. Ag NP coating has consistently been shown to exhibit activity against adherent bacteria within in vitro culture in addition to suspended planktonic bacteria. Notably, several studies have revealed that Ag NPs can induce the downregulation of biofilm-forming genes *icaA*, *icaD*, *fnbA*, and *fnbB*, alluding to an additional mode of action in tandem with their conventional membrane and intracellular protein disruption [51,202]. Of note, while most the studies validated a dose-responsive in vitro antibacterial effect, a balance had to be achieved in all cases to preserve human cell viability; this highlights that even with surface coating to avoid systemic toxicity, Ag NPs can potentially trigger local cell death in situ. Moreover, human cell attachment to the surface should be shown in addition to simple cell viability studies, given that the patient’s cells effectively compete with MRSA to colonise implant surfaces [185]. For example, Wang and colleagues showed experimentally that fibroblasts preferentially bind and cover the Ag NP-coated material over MRSA in a co-culture assay (Figure 7; [51]). Future studies that employ similar in vitro co-culture assays to provide additional information about a coating’s safety and efficacy in a more physiologically-representative model are welcomed by the authors.

Apart from silver-based nanoparticulate coatings, other nanomedicine coatings that have been investigated to prevent or eradicate biofilms include copper, silicon nitride, chitosan, immobilized AMPs, synthetic nanoparticles, and liposomes (Table 5). Copper has been investigated in its own right as a coating for titanium or as a replacement in alloys [208,209]; as a nanoparticle coating, it can elicit MRSA anti-biofilm activity with the advantage of less toxic side effects than silver [210]. Other metal ion coatings under examination include titania nanoparticles, as derived from the metal that is so often the substrate underneath other coatings [211]. Lopes et al. studied their antibacterial potential as part of a diamond-like carbon surface coating and verified their in vitro activity against both planktonic and sessile MRSA. Chitosan-based nano-coatings have also exhibited such activity, although the natural polymer has primarily served as a delivery vehicle for other therapeutics, rather than as a coated antimicrobial in its own right [212,213,214]. Notably, several studies have applied various combinations of polymers, drugs, and manufacturing techniques to develop multi-drug nanomedicine coatings that have shown anti-biofilm potential within in vitro and in vivo models [215,216,217]. While these data yield positive findings for the prevention of MRSA biofilms, the translation of a medical device that incorporates such a multitude of components into the clinic can present a significant regulatory hurdle that needs to be overcome, as discussed in Section 5.

## 5. Future Perspectives: Challenges, Opportunities, and the Path for Clinical Translation

### 5.1. Challenges for Nanomedicine and MRSA Management

In spite of the abundance of preclinical research that has been outlined in this review, there are few clinical trials for the use of nanomedicines in MRSA management underway [218]. Specifically, one completed trial has compared conventional central venous catheters to silver nanoparticle-loaded catheters, although no results have been published to date (NTC00337714). In general, apart from the particular toxicity issues that have been discussed in previous sections, nanomedicines for MRSA therapy face the same challenges as in any other nanomedicine application—standardized preclinical in vitro testing and animal models, as well as efficient and reproducible scale-up for industrial manufacture [219]. (In keeping with this review, MRSA-specific translation challenges will be discussed; for further information concerning nanomedicine scale-up in general, the reader is referred to other reviews in this area [220,221]).

In order to progress any novel nanomedicine therapies into clinical trials and beyond, it is critical to standardize the in vitro analyses that are required to reach this point, particularly from a regulatory point of view. Over the course of this review, a plethora of different methods for cell viability, adhesion, toxicity, nanoparticluate characterization, and drug activity have been performed; this ranges from assays, such as Live/Dead^®^ staining, proliferation assays [222], and biofilm imaging. While the majority of these methods have functioned adequately within each study, the identification of key preclinical criteria and harmonization of protocols to them can facilitate the streamlined progression of promising nanomedicines to the next stage of drug development. Indeed, a degree of heterogeneity exists even in the selection of the bacterial strain of MRSA, and as alluded to in Section 1, it is advisable to design a study with the target strain of MRSA in mind for a particular target population or community [15,18]. Additionally, the selection of an appropriate animal model that matches the particular clinical MRSA case is paramount, with different models available for soft tissue, septic, catheter-related, or osteomyelitis infection [223,224,225]. Once these essential issues have been resolved, we predict that a clearer pathway for clinical translation will present itself and a greater number of the innovative projects outlined in this review will have the potential to progress to meet the challenge of MRSA.

### 5.2. Opportunities for Nanomedicine and MRSA Management

Several opportunities exist for the field of nanomedicine to succeed in the path to clinical translation for MRSA management. These include a pathway for approved nanomedicines in other clinical applications, public investment in the development of novel antimicrobials to combat drug resistance, and industry incentives to produce new antimicrobials.

At present, approximately 26 nanomedicines are clinically approved for systemic administration, with almost 50 more in clinical trials [219]. They are licensed for indications such as cancer, iron replacement, image contrast agents, vaccination, anaesthesia, fungal infection, and macular degeneration. The regulatory approval of these medicines provides an opportunity for MRSA nanomedicines through the provision of a regulatory approval template that can be applied to the development of similar therapeutics within the field of antimicrobials. Of note, however, is the fact that the majority of the approved nanomedicines are liposomal or colloidal in nature, which might not exactly correlate to the regulatory demands of metal-based nanoparticles. Although the FDA states that the approval of nanomedicines that are used in this context will largely be the same process as for any other medicinal product, we expect that as the number of approved nanomedicines increases across the market, industry appeal for antibacterial nanomedicines will expand thereafter. Of additional interest to the pharmaceutical industry in this space are multiple international and national initiatives to incentivize research, development, and translation of novel antimicrobial therapies to address antibacterial drug resistance [226]. These incentives, coupled with the drive to repurpose currently-approved drugs as antimicrobials [123,227,228], present specific opportunities for fostering industry interest in MRSA nanomedicines and potentially academic-industry collaboration to carry promising therapies forward to market. Overall, as reflected by the WHO report [1] and continued presence in the mainstream media, antimicrobial continues to be a global issue with a high degree of public attention, facilitating the continued impetus for invested interest in developing new treatments to eradicate multidrug resistant bacteria, such as MRSA.

### 5.3. Conclusions

While the threat posed by MRSA infections can never be underestimated, the information contained in this review highlights the significant progress being made in developing the next generation of therapeutics. Specifically, in developing new active substances that will circumvent the traditional resistance mechanisms of MRSA, as well as breathing new life into pre-existing and previously approved drugs. Taken together with the efforts now being undertaken in addressing the obstacles posed by biofilm formation, it is certain that new treatments for MRSA infections will begin the journey towards clinical trials and regulatory approval in the near future. However, the need for consensus and standardization in assessing anti-MRSA nanomedicine candidates remains undiminished. This will allow for a more open and cross-comparable environment, which will ultimately benefit both the researcher and the patient.

## Figures and Tables

**Table 1:** Mechanism of action and development of metal nanoparticles (NP)-based strategies for Methicillin-resistant *Staphylococcus aureus* (MRSA) treatment.

Type	Mode of Action	MICs Reported	Biofilm Effective?	Development Status	Delivery Methods	References
Ag NPs	Bacterial cell membrane disruption Cytochrome and electron transport inhibition DNA/RNA binding and inhibition of replication Ribosomal binding and inhibition of protein synthesis ROS formation Inhibits gram + cell wall formation	0.25–64.5 μg/mL	Yes	In vitro/in vivo validated	Micro-Patterned on titanium implants Topical hydrogel	[32,33,40,41,51,52]
ZnO NPs	Bacterial cell membrane disruption ROS formation	1–10 μg/mL	Yes	In vitro/in vivo validated	Intradermal I.V injection	[53,54,55,56,57]
Cu/CuO NPs	Interacts with amine and carboxyl groups on bacterial cell surface ROS formation	1.87 μg/mL–1 mg/mL	Yes	In vitro validated	n/a	[44,58,59,60,61]
TiO_2_ NPs	ROS formation following UV stimulation (photocatalysis) UV-independent effects (mechanism unknown)	100 μg/mL–15 mg/mL	Low activity, rarely tested	In vitro validated	n/a	[62,63,64,65,66]
MgX2/MgO NPs	MgX2 enzymatic inhibition ROS formation MgO-induced halogen adsorption	1.5 mg/mL	Yes	In vitro/in vivo validated	coated on titanium implants (osteomyelitis model)	[67,68,69,70]
Au NPs	No intrinsic antimicrobial effect Activity achieved through functionalization or combination therapy	8–32 μg/mL (modification dependent)	Yes (combination therapy)	In vitro/in vivo validated (combination therapy)	Systemic sepsis	[46,71,72,73,74,75,76]
Bi NPs	Radiation-stimulated free radical formation and DNA damage	0.2–11.47 µM	Yes	In vitro validated	n/a	[77,78,79,80]

MIC: Minimum Inhibitory Concentration, Ag NPs: Silver Nanoparticles, ZnO: Zinc Oxide, I.V: intravenous, Cu/CuO: Copper/Copper Oxide, TiO_2_: Titanium Oxide, MgX2: Magnesium with X_2_ referring to a bonded halide, Au: Gold, Bi: Bismuth.

**Table 2:** Overview of MRSA nanomedicines using AMPs and peptidomimetics.

Type	Mode of Action	Biofilm Effective?	Development Status	Delivery Methods	Outcomes	Refs.
Modified-RIP peptides	Inhibition of the RNAIII-activating protein (RAP) Disruption of quorum sensing in MRSA	Yes	In vitro/in vivo validated	UTI model/Sepsis model I.P injection	Significant decreases in bacterial counts. In vivo activity comparable to vancomycin	[91,92]
RR (WLRRIKAWLRR) RRIKA (WLRRIKAWLRRIKA)	Bacterial membrane disruption	Yes	In vitro validated	n/a	RR MIC = 12–24 μg/mL RRIKA MIC = 3.7–7.4 μg/mL	[97]
Myxinidin2 (KIKWILKYWKWS) Myxinidin3 (RIRWILRYWRWS)	Membrane disruption via binding to lipoteichoic acid (LTA)	Yes	In vitro validated	n/a	Myxinidin2 MIC = 6.7–26.8 μg/mL Myxinidin3 MIC = 7.16 μg/mL	[84]
Synthetic Peptidomimetics	Hydrocarbon tail length and functional group specific Intracellular-targeting and disruption	Not-tested	In vitro validated	n/a	MIC = 4–32 μg/mL	[90]
Synthetic Peptidomimetics	Bacterial membrane disruption	Yes	In vitro validated	n/a	MIC= 1.7–454 μg/mL (conformation dependent)	[98]
PA-28 (modified TAT, C16-W-I-L-A2-G3-K9-TAT)	Bacterial membrane disruption	Not-tested	In vitro/in vivo validated	S. *aureus*- induced meningitis I.V delivery	MIC = 147 μg/mL In vivo efficacy comparable to vancomycin	[99]
CG3R6TAT	Bacterial membrane disruption	Not-tested	In vitro/in vivo validated	S. *aureus*- induced meningitis I.V delivery	MIC = 35.667 μg/mL In vivo efficacy comparable to vancomycin	[100]
IDR-1 (KSRIVPAIPVSLL) IDR-1002 (VQRWLIVWRIRK)	chemokine induction and reduction of pro-inflammatory cytokines	Not-tested	In vitro/in vivo validated	Mice pre-treated with IDRs (I.P) prior to MRSA	No direct MIC Significant MRSA reductions	[101,102]

UTI: Urinary tract infection, I.P: Intra-peritoneal, IDR: Innate defense–regulator.

**Table 3:** Enhancing anti-MRSA effect of antibiotics via encapsulation in chitosan nanoparticles.

Chitosan Characteristics	Co-Delivery of	Biofilm Effective?	Development Status	Delivery Methods	Outcomes	Refs.
Mw = 1.3–4 kDa %DD = 98% (solution only)	Erythromycin/ tilmicosin	Yes	In vitro/in vivo validated	Intra-mammary injection in mice and cows	Co-delivery enhanced MIC of erythromycin 4-fold (0.12 µg/mL) Significant decreases in MRSA using co-delivery in vivo	[126]
Mw = 107 kDa %DD = 75–85%	No co-delivery	Yes	In vitro validated	n/a	MIC of 1.25 mg/mL	[129]
Low Mw	Ceftriaxone	Not tested	In vitro/in vivo validated	Neutropenic mouse thigh model	ZOI 28 mm vs. ≤17 mm (blank NPs) vs. 0 mm (free ceftriaxone) Up to 41% decreases vs. controls in vivo but non-significant	[130]
Medium Mw	Amoxicillin	Not tested	In vitro validated	n/a	MIC = 6.1 µg/mL vs. ≤32 µg/mL (blank NPs) vs. 8 µg/mL (free Amoxicillin)	[134]
O-Carboxymethyl chitosan Mw = 12 kDa %DD = 61.8%	Tetracycline	Not tested	In vitro validated	n/a	Intracellular MRSA survival 2.5% in encapsulated vs. 15% using free tetracycline	[135]
Medium Mw 190–310 kDa %DD = 75–85%	Vancomycin	Not tested	In vitro/in vivo validated	Rat osteomyelitis model	Chitosan-vanco = 3354 ± 3366 CFU/g IM injection of vanco = 52,500 ± 25,635 CFU/g Control = 68,750 ± 16,637 CFU/g	[131]
Not stated, folate tagged	Vancomycin	Yes	In vitro validated	n/a	MIC decreased 97.52% using nanoparticle vancomycin	[132]
Low Mw, %DD = 75–85%, anionic gemini surfactant (AGS) modified	Vancomycin	Not tested	In vitro/in vivo validated	Mouse skin model	In vivo MRSA clearance was 8-fold higher in nanoparticle treated animals	[133]
Low Mw, %DD = 75–85%	Streptomycin Ampicillin	Not tested	In vitro validated	n/a	Controlled release and theranostic potential but reduced anti-microbial effect	[136,137,138]

Mw: Molecular Weight, %DD: % Deacetylation, ZOI: Zone of inhibition, IM: Intra-muscular.

**Table 4:** Silver nanoparticle coating of surfaces to prevent biofilm formation.

Nanoparticle Coating	Status	Outcome(s)	Refs.
*Ag-TiO_2_*			
AgNO_3_ coating of nanotubes	In vitro & in vivo validated	Activity against planktonic and adherent MRSA up to 30 days in vitro Antibacterial activity & biocompatibility up to 4 weeks in vivo	[40,201,202]
AgCl-TiO_2_ coating from AgCl-TiCl_4_ sol reaction	In vitro validated	Inhibtion of *S. epidermidis* biofilm formation	[204]
PEO TiO_2_ coating with silver acetate HMC	In vitro validated	Activity against planktonic & sessile *S. epidermidis* and MRSA Downregulation of *ica* & *fnb* genes Preferential adherence of fibroblasts in co-culture	[51]
*Ag-Ti*			
Plasma immersion ion implantation	In vitro & in vivo validated	Embedded NPs less toxic than free NPs 60-day *S. epidermidis* biofilm reduction Downregulation of *ica* genes	[203]
SLM with ALD of silver nanolayer	In vitro & in vivo validated	Reduced *S. epidermidis* adherence & growth in vitro Indication of slow MRSA growth in vitro In vivo bone ingrowth and biocompatibility	[205]
*Other AgNPs*			
AgO-HA sprayed Ti surface	In vitro & in vivo validated	Reduced MRSA biofilm coverage over 14 days in vitro & in vivo	[206]
Ag-DLC-PE immersion ion implantation	In vitro validated	Reduced *S. epidermidis* planktonic growth over 24 h	[207]

ALD: Atomic layer deposition; DLC-PE: Diamond-like carbon-coated polyethylene; HA: Hydroxyapatite; HMC: Hydrothermal metal coupling; PEO: Plasma electrolytic oxidation; SLM: Selective laser melting.

**Table 5:** Alternative nanomedicine surface coatings to silver for prevention of biofilm formation.

Nanomedicine Coating	Status	Outcome(s)	Ref.
*Other metal ions*			
CuNPs in polyglycerol coating	In vitro validated	Activity against MRSA in planktonic and biofilm form Non-toxic to murine fibroblasts*o*	[210]
TiO_2_- & HA-loaded DLC film produced by PECVD	In vitro validated	Reduced *S. aureus* planktonic growth over 24 h	[211]
*Chitosan*			
AgNP-loaded chitosan-HyA layer-by-layer coating on Ti_3_	In vitro validated	Inhibtion of *S. aureus* biofilm formation after 7 days 14-day activity against planktonic and adhered *S. aureus*	[212]
Minocycline-loaded chitosan-alginate multilayer coating on Ti_3_	In vitro validated	Inhibtion of *S. aureus* biofilm formation after 7 days 14-day activity against planktonic and adhered *S. aureus*	[213]
Tetracycline-loaded chitosan-gelatin NPs on Ti_3_	In vitro & in vivo validated	Non-toxic to murine pre-osteoblasts at 7 days Inhibtion of *S. aureus* growth in vivo after 7 days Reduction in white blood cell count after 7 days	[214]
*Mutli-drug release*			
Vancomycin & AgNP-coated TiO_2_ nanotubes	In vitro & in vivo validated	Activity against planktonic and sessile MRSA from 1 h to 28 days Preferential adherence of fibroblasts in co-culture Reduced MRSA adherence and infection in vivo after 15 days	[215]
PLGA-PCL nanofibre coating of Ti with (i) vancomycin & rifampicin (ii) linezolid & rifampicin (iii) daptomycin & rifampicin	In vitro & in vivo validated	Vancomycin & rifampicin exhibited the greateast activity in vitro against planktonic *S. Aureus* All combinations prevented biofilm formation in vivo	[216]
Naproxen & AgNP-loaded PVA-chitosan coating of Ti	In vitro validated	Activity against planktonic *S. Aureus* Indication of slow MRSA growth in vitro Biocompatiblity with human osteoblasts	[217]

DLC: Diamond-like carbon; HA: Hydroxyapatite; HyA: Hyaluronic Acid; PCL: Polycaprolactone; PECVD: Plasma-enhanced chemical vapor deposition; PLGA: Polylactic-*co*-glycolic acid; PVA: Polyvinyl alcohol.
